# Supplementary material for: Molecular Hydrogen Improves Rice Storage Quality via Alleviating Lipid Deterioration and Maintaining Nutritional Values
Source: Plants (Basel). 2022 Sep 30;11(19):2588. doi: 10.3390/plants11192588 (PMC9571184; doi:10.3390/plants11192588)
Supplement: Supplementary file 1 [file plants-11-02588-s001.zip › plants-1908744-supplementary.pdf]

Supplementary Information

Molecular hydrogen improves rice storage quality via alleviating lipid deterioration and maintaining nutritional values

Chenxu Cai, Zhushan Zhao, Yingying Zhang, Min Li, Longna Li, Pengfei Cheng, Wenbiao Shen*

College of Life Sciences, Laboratory Center of Life Sciences, Nanjing Agricultural University, Nanjing 210095, China

*Corresponding author: Wenbiao Shen

E-mail: wbshenh@njau.edu.cn

Tel: +86-025-84399032;

Table S1 Primers of qPCR used in this study

Gene	Forward primer (5’-3’)	Reverse primer (5’-3’)
OsActin (*45*)	CACGTGAGCAAGGCTCTTTTG	GGCATCCCCCTCCTCATAGA
OsUBIQUITIN (*46*)	GACGGACGCACCCTGGCGAACTAC	TGCTGCCAATTACCATATACCACGAC
OsLOX1 (XM_015771289)	TTCTTCACCGTCAAGTCGT	TCCGAGAACTGCTCGTC
OsLOX2 (*48*)	TACAAGTCCGACGAGGAGGT	CCACATGATGGTGGTCAAGA
OsLOX3 (*49*)	TCGCATTGGCTCGCACCCT	TCGTCTTCTTCAGCCGCACGAT

OsActin (reference gene), beta-actin; *OsUBIQUITIN (reference gene), ubiquitin*; *OsLOX1, 1ipoxygenase 1*; *OsLOX2, 1ipoxygenase 2; OsLOX3, 1ipoxygenase 3.*

48. Yang, J.; Wang, Y.; Liu, L.; Liu, L.; Wang, C.; Wang, C.; Li, C. Effects of exogenous salicylic acid and pH on pathogenicity of biotrophy-associated secreted protein 1 (BAS1)-overexpressing strain, *Magnaporthe oryzae*. *Environ. Sci. Pollut. Res.* **2018**, *26*, 13725–13737. https://doi.org/10.1007/s11356-018-2532-y.

49. Xu, H.; Wei, Y.; Zhu, Y.; Lian, L.; Xie, H.; Cai, Q.; Chen, Q.; Lin, Z.; Wang, Z.; Xie, H.; et al. Antisense suppression of *LOX3* gene expression in rice endosperm enhances seed longevity. *Plant Biotechnol. J.* **2014**, *13*, 526–539. https://doi.org/10.1111/pbi.12277.

Table S2 Profiles of volatiles in one year stored rice (Huruan 1212). Concentrations of volatile compounds were expressed as μg kg^{-1} of rice fresh weight, equivalent to 2,4,6-trimethylpyridine (TMP). Values were mean ± SD. ND: not detected; Con: Control, irrigated with ditch water; HNW: irrigated with hydrogen nanobubble water.

Family	Number	Compound	MW	MF	Compound CID	Content (μg kg^{-1})	
						Con	HNW
Aldehydes	1	Pentanal	86.13	$C_5H_{10}O$	8063	19.77±2.68	6.62±0.77
	2	Hexanal	100.16	$C_6H_{12}O$	6184	270.10±19.03	183.71±1.40
	3	Heptanal	114.19	$C_7H_{14}O$	8130	23.68±1.86	12.73±0.68
	4	Benzaldehyde	106.12	C_7H_6O	240	17.62±1.18	32.25±1.55
	5	Octanal	128.21	$C_8H_{16}O$	454	17.30±2.49	11.56±0.57
	6	(E)-2-Octenal	126.20	$C_8H_{14}O$	5283324	20.02±0.79	18.99±1.48.
	7	Nonanal	142.24	$C_9H_{18}O$	31289	74.98±2.97	106.8±6.05
	8	(E)-2-Nonenal	140.22	$C_9H_{16}O$	5283335	12.84±0.39	9.47±2.08
	9	Decanal	156.26	$C_{10}H_{20}O$	8175	22.36±1.05	28.89±2.74
	10	Undecanal	170.29	$C_{11}H_{22}O$	8186	3.51±0.13	5.53±0.12
	11	2-Butyl-2-octenal	182.30	$C_{12}H_{22}O$	5362697	5.43±0.50	3.01±0.25
Alcohols	1	Ethanol	46.07	C_2H_6O	702	ND	3.84±0.38
	2	1-Pentanol	88.15	$C_5H_{12}O$	6276	22.05±2.48	24.68±1.57
	3	1-Hexanol	102.17	$C_6H_{14}O$	8103	82.58±11.22	98.49±4.08
	4	1-Heptanol	116.20	$C_7H_{16}O$	8129	7.37±0.57	10.09±0.82
	5	1-Octen-3-ol	128.21	$C_8H_{16}O$	18827	35.92±1.34	21.88±0.88
	6	Cis-linalool oxide	170.25	$C_{10}H_{18}O_{22}$	6428573	6.21±0.27	8.03±0.09
	7	Terpinen-4-ol	154.25	$C_{10}H_{18}O$	11230	3.68±0.06	2.28±0.05
	8	*p*-Menthan-1-ol	156.26	$C_{10}H_{20}O$	89437	13.94±1.73	9.16±0.12
Ketones		Acetone	58.08	C_3H_6O	180	10.34±1.14	15.22±0.54
	2	2-Heptanone	114.19	$C_7H_{14}O$	8051	46.60±1.46	34.98±1.06
	3	4-Methyl-cyclohexanone	112.17	$C_7H_{12}O$	11525	9.65±0.9	6.96±0.60

Ketones	4	6-Methyl-5-hepten-2-one	126.20	$C_8H_{14}O$	9862	12.93±1.16	24.80±1.68
	5	3-Octen-2-one	126.20	$C_8H_{14}O$	5363229	3.38±0.19	3.38±0.16
	6	(R,S)-5-Ethyl-6-methyl-3E-hepten-2-one	154.25	$C_{10}H_{18}O$	146160920	12.42±1.33	7.42±0.34
	7	6,10-Dimethyl-2-undecanone	198.34	$C_{13}H_{26}O$	95495	3.52±0.12	3.05±0.02
	8	Geranylacetone	194.31	$C_{13}H_{22}O$	1549778	11.86±0.80	17.06±0.41
	9	6,10,14-trimethylpentadecan-2-one	268.50	$C_{18}H_{36}O$	10408	4.23±0.58	2.47±0.17
Esters	1	Formic acid, octylester	158.24	$C_9H_{18}O_2$	8176	19.10±1.66	20.47±0.18
	2	Dodecyl acrylate	240.38	$C_{15}H_{28}O_2$	75084	17.17±1.56	12.98±0.61
	3	Sulfurous acid, octadecyl 2-propyl ester	376.60	$C_{21}H_{44}O_3S$	6420358	7.74±0.66	7.61±0.17
	4	Octadecanoic acid, 2-oxo-, methyl ester	312.50	$C_{19}H_{36}O_3$	620002	5.71±0.18	5.65±0.14
	5	Dimethyl phthalate	194.18	$C_{10}H_{10}O_4$	8554	5.81±0.41	8.43±0.48
Others	1	Acetic acid	60.05	$C_2H_4O_2$	176	16.20±5.19	9.59±0.52
	2	Ethylbenzene	106.16	C_8H_{10}	7500	3.32±0.58	9.61±1.09
	3	Undecane	156.31	$C_{11}H_{24}$	14257	3.80±0.26	4.25+0.06
	4	2-Aminomethyl-5-methylamino-1,3,4-oxadiazole	128.13	$C_4H_8N_4O$	581786	4.29±0.33	5.22±0.18
	5	Tridecane	184.36	$C_{13}H_{28}$	12388	4.61±0.17	6.54±0.48
	6	5-pentyloxolan-2-one	156.22	$C_9H_{16}O_2$	7710	8.21±0.76	10.34±0.47
	7	Hexadecane	226.44	$C_{16}H_{34}$	11006	3.48±0.18	3.05±0.57

Table S3 Profiles of hydrolyzed amino acids in stored rice (Huruan 1212). Contents were expressed as mg g^{-1} of rice dry weight. Values were mean ± SD. Con: Control, irrigated with ditch water; HNW: irrigated with hydrogen nanobubble water.

**Type**	**Content（mg g^{-1}）**	
	**Con**	**HNW**
Threonine	2.11±0.10	2.65±0.06
Valine	2.25±0.70	3.51±0.05
Methionine	0.29±0.13	0.27±0.06
Isoleucine	1.88±0.31	2.36±0.11
Leucine	4.18±0.15	4.95±0.10
Phenylalanine	2.76±0.09	3.40±0.17
Histidine	1.54±0.02	1.84±0.02
Lysine	2.41±0.05	2.80±0.09
**Total**	**17.44±1.21**	**21.80±0.28**
Aspartic acid	6.07±0.06	6.89±0.15
Serine	2.67±0.20	3.39±0.09
Glutamate	11.46±1.14	14.12±0.11
Glycine	3.30±0.14	3.68±0.02
Alanin	3.70±0.35	4.29±0.07
Cystine	0.99±0.28	0.68±0.03
Tyrosine	2.08±0.02	2.60±0.06
Arginine	5.15±0.21	6.24±0.34
Proline	4.43±1.47	3.94±0.05
**Total**	**39.89±0.80**	**45.84±0.59**

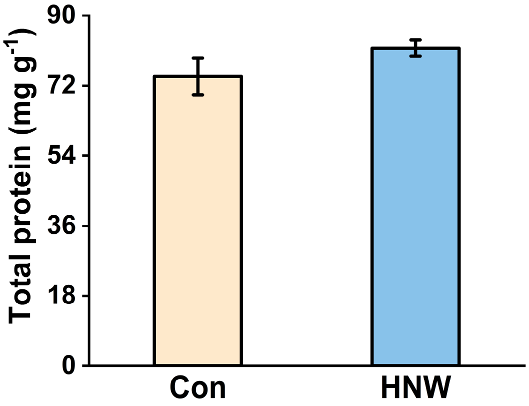

Figure S1 Profiles of total protein contents from different treatments in stored rice. Values are means ± standard deviation (SD) of three biological repetitions.

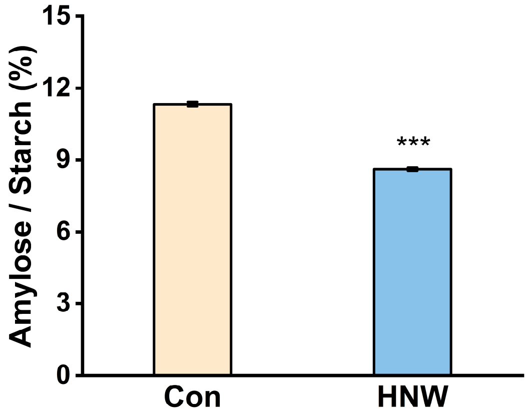

Figure S2 Ratio of amylose in starch from different treatments in stored rice. Values are means ± standard deviation (SD) of three biological repetitions. *** indicate significant difference (*t*-test, $P < 0.001$).

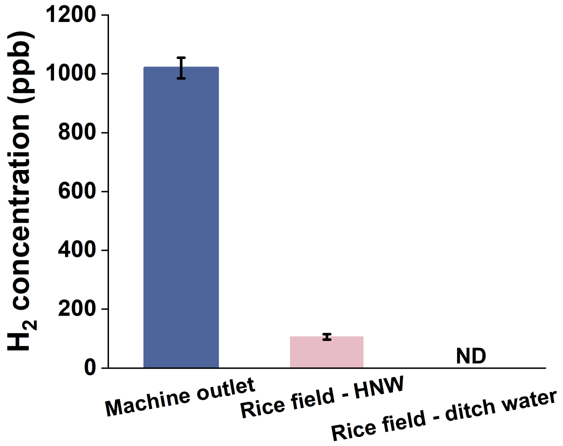

Figure S3 Concentration of H_2 in the machine outlet and fields with different treatments (measured on 27 June 2020). Values are means ± standard deviation (SD) of six repetitions in each field randomly. ND: not detected.

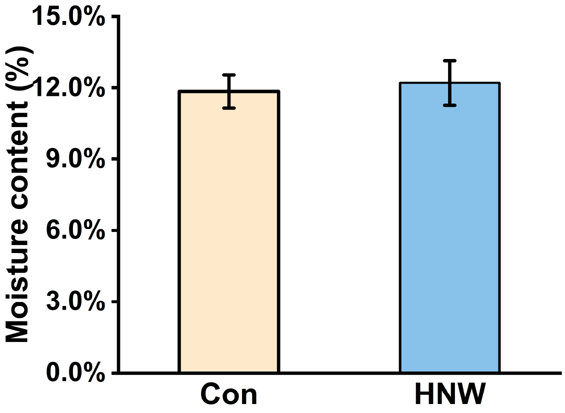

Figure S4 Profiles of moisture content from different treatments in stored rice. Values are means ± standard deviation (SD) of three biological repetitions.
